# Disrupting the Systems: Opportunities to Enhance Methodological Approaches to Address Socio-Structural Determinants of HIV and End the Epidemic Through Effective Community Engagement

**DOI:** 10.1007/s10461-021-03475-7

**Published:** 2021-10-07

**Authors:** Carlos E. Rodriguez-Diaz, Wendy Davis, Marcia V. Ellis, Martha Sichone Cameron, Yeycy Donastorg, Lisa Bowleg, Alan Greenberg, Deanna Kerrigan

**Affiliations:** 1 Department of Prevention and Community Health, Milken Institute School of Public Health, George Washigton University, 950 New Hampshire Ave NW, Suite 300, Washington, DC 20052 USA; 2 DC Center for AIDS Research, Washington, USA; 3 International Community of Women Living With HIV, North America, London, UK; 4 Instituto Dermatológico y Cirugia de Piel, Santo Domingo, Dominican Republic; 5 Department of Psychology and Brain Sciences, George Washington University, Washington, USA; 6 Department of Epidemiology, George Washington University-Milken Institute School of Public Health, Washington, USA

**Keywords:** HIV, Social determinants of health, Methodologies

## Abstract

A world without HIV is only possible by addressing the socio-structural determinants of health. Our understanding of socio-structural determinants is constantly changing, and parallel changes must occur with the methodologies used to explain the drivers of the HIV epidemic. We argue for the need to engage communities in the planning, implementation, and dissemination of research on the socio-structural determinants of HIV. Community engagement should cross-cut various types of research including rigorous measurement development of socio-structural determinants and novel analytic techniques to model their role in the trajectory of the epidemic and the impact of interventions. Considering the role of place, we recommend collaboration between scientists and communities in the interpretation of results from studies that map HIV-related behaviors and movement. As we collectively delve into historically oppressive systems with colonial antecedents, we must be ready to challenge these systems and replace them with collaborative models. The success of research-driven HIV policy and programming will best be evaluated with methodologies derived from the insights of the very individuals that these policies and programs aim to serve.

## Resumen

Un mundo sin VIH es posible sólo si atendemos los determinantes socio-estructurales de la salud. Nuestra comprensión sobre determinantes socio-estructurales cambia constantemente y cambios similares deben ocurrir en las metodologías utilizadas para explicar los factores que rigen la epidemia del VIH. Argumentamos sobre la necesidad de involucrar las comunidades en la planificación, implementación y diseminación de investigaciones sobre los determinantes socio-estructurales del VIH. La participación comunitaria debe ser transversal en varios tipos de investigaciones, incluyendo el desarrollo riguroso de métricas sobre los determinantes socio-estructurales y técnicas noveles para la modelación de su rol en las trayectorias de la epidemia y el impacto de intervenciones. Considerando el rol que tiene el lugar físico, recomendamos la colaboración de científicos y comunidades en la interpretación de resultados de estudios que crean mapas de las conductas relacionadas al VIH y la movilidad de las personas. En la medida en que examinamos sistemas históricamente opresivos con antecedentes coloniales, debemos estar listos para retar estos sistemas y remplazarlos con modelos colaborativos. El logro de políticas y programas de VIH informados por la investigación sería evaluado mejor si se utilizan metodologías guiadas por el conocimiento de las personas a las cuales estas políticas y programas persiguen servir.

## Introduction

Substantive progress towards ending the HIV epidemic globally and domestically rests on addressing socio-structural determinants of health and the barriers to preventive care and access to care [1, 2]. This is particularly true for key populations who comprise 95% of the new HIV infections worldwide [2]. Since very early on in the HIV epidemic, public health scientists have documented how social factors like stigma, heterosexism, racism, and poverty drive inequitable rates of HIV infections and deaths in communities historically marginalized by structural inequality [3]. More recently, it has been demonstrated that socio-structural factors such as low educational attainment, incarceration, unstable housing, and policies that deterred harm reduction increased the likelihood of HIV infection and disease progression in populations made socially vulnerable [4–6]. As our lexicon of socio-structural determinants expands, so must the breadth, precision, and sophistication of the tools we use to elucidate their complex interplay on various health outcomes. Indeed, progress in measuring and modeling how social-structural factors drive the HIV epidemic has mirrored our increasing appreciation of the range and depth of these factors.

The work described in this supplement, which highlights innovation in mathematical and statistical modeling, geographical and social mapping, and the measurement and documentation of nuanced social constructs, reflects essential steps in this progress. Simultaneously, the need for continued methodological innovation, and specifically innovation driven by meaningful partnership and collaboration between members of any given community that must interact with systems of HIV prevention and care, and social and behavioral scientists and practitioners, is clear. Successful interventions aimed at addressing the global HIV pandemic must be founded in theoretical frameworks and methodological tools that fully reflect and respond to the lived experience of affected communities. Further, research and actions on the social and structural determinants of HIV should place partnerships with community members at the forefront. To help define this critical path, we reflect on the benefits and challenges that remain in grounding methodological innovations in a social context and thoroughly engaging the community in the conceptualization, operationalization, and utilization of novel methods around the socio-structural determinants of HIV as revealed through this volume’s collection of research.

Methods used by studies conducted through the Request for Applications (RFA-MH16-200 and RFA-MH16-205) and featured in this supplement can be grouped into three primary approaches: modeling, mapping, and measuring. In this commentary, we consider opportunities for an integrated and collaborative approach within each of these methodological approaches.

## Models

Models serve efforts to effectively combat the HIV epidemic in fundamental and often highly efficient ways. Modeling helps governments and other donors determine how best to apportion limited resources to impact incidence, morbidity, or mortality significantly. Models of the HIV epidemic have traditionally considered the impact of the roll-out of different biomedical prevention or treatment interventions. Still, they have been less likely to model the effect of addressing socio-structural determinants of HIV. In this volume, Jahagirdar et al. [7] took an important step towards acknowledging the potential importance of social determinants in modeling the HIV epidemic. In particular, they look at the impact of education and exposure to sexual violence on HIV incidence. They found that education years per capita contributed the most to explaining the observed variation in HIV incidence. For example, a 1-year increase in mean education years was associated with a − 0.35 (− 0.39; − 0.31) percent decline in the HIV incidence rate [7]. The authors note that their work suggests that an investment in biomedical interventions such as access to ART alone is not enough and may be less effective than addressing social and structural factors such as access to education and sexual violence.

Modeling such as this, which pulls from comprehensive country-level data, offers valuable additional insights into the role of social determinants in cross-national differences in HIV incidence and mortality. At the same time, the impact of this work could be further strengthened by closer partnerships with community stakeholders. Such partnerships could guide both the selection of data sources for modeling and the interpretation of results. For example, while community partners might find access to education compelling, they might suggest other model inputs such as the presence of laws that criminalize and stigmatize specific populations, certain features of health care access or cost or economic opportunity. Social determinants operate at multiple levels, and community stakeholders are well poised to offer guidance on which factors at which level, including national, regional, and local, may be most impactful on epidemic progression. Community stakeholders can also provide valuable insight into why a factor may or may not be having a statistically significant impact on HIV incidence. How a factor is measured and the degree to which it authentically reflects context and community reality, for example, may obfuscate its actual contribution to HIV outcomes. Finally, community partners can offer critical guidance on how best to disseminate modeling results. They are often intricately engaged in national, regional, and local networks and are particularly well-positioned to help communicate findings in ways that are accessible and actionable to community members, government partners, and other stakeholders.

In a very different type of model, one that looks across individuals instead of across settings, Stoner et al. were able to consider how HIV incidence might be modified if attention was given to addressing several different social determinants at once [8]. In particular, they model the potential impact of a monthly cash transfer to adolescent girls and young women (AGYW) living in South Africa in concert with efforts to increase “parental care” (defined as “How much do you feel that (parent/guardian) cares for you”, and mental health. The model constructed by Stoner et al. showed that combining a monthly cash grant with interventions to increase parental care and reduce depression in AGYW could substantially reduce HIV incidence for AGYW above the provision of cash alone [RD − 3.0%; (95% CI − 5.1%, − 0.9%)] [8]. This modeling exercise demonstrates another significant utility of models as we work to understand how best to address social determinants in the context of multi-pronged interventions. In this instance, Stoner et al. [8] explained why an intervention alone, in this case, cash transfer, while promising, may not demonstrate consistent efficacy and how this can be alleviated.

As with the type of modeling employed by Jahagadir et al. [7], the approach used by Stoner et al. [8], can also be strengthened through additional collaboration with community stakeholders. For example, stakeholders can explain why parental care and depression may be centrally crucial in whether or not a cash transfer program is efficacious. In future modeling efforts, community stakeholders with on the ground vision and expertise may offer inputs into other potential facilitators or barriers to structural interventions such as cash transfer. Finally, as noted earlier, community partners with their local, regional, and governmental networks and deep understanding of opportunities within programs are well-positioned to advise academic partners on how best to present and implement findings from models such as these.

## Maps

As conceptual models of the social determinants of HIV mature, there is an increasing appreciation of the central importance of place in the expression of social determinants. Place is foundational to the social determinants of HIV, driving both an individual’s exposure to risk as well as their access to HIV prevention and care. Two studies in this volume apply methodological innovation to the concept of place and assessments of its impact on HIV risk and incidence. Madden and colleagues used an ecological momentary assessment (EMA) protocol that included a daily retrospective survey to collect data on sexual and substance use risk among homeless and formerly homeless young adults [9]. Data collected in real-time from participants’ smartphones found that individuals currently experiencing homelessness had 3.23 times (95% CI 0.98–10.65) higher odds of reporting exchange sex in the 7-day measurement period than those who were formerly homeless and now residing in housing programs (*p* = 0.054) and that all participants, regardless of whether or not they were homeless, who had sexual intercourse under the influence of drugs or alcohol had a much higher odds (OR 14.24; 95% CI 3.81–53.25) of reporting condomless sex during the study week (*p* < 0.001) [9]. Through this work, Madden et al. illuminate the real-time connection between place and risk and context and risk and underscore the vulnerability ascribed to young adults by homelessness, a social determinant of HIV.

Assessing both the risk that individuals encounter in real-time and the context within which that risk occurs is an important methodological development in studying social and structural determinants. At the same time, this research raises important questions and opportunities. For example, in this study, which took place over 1 week, Madden and colleagues [9] found relatively little overall sexual activity, with 26.7% of the sample reporting any sexual intercourse in the last 7 days. Community representatives can offer insights as to why this may have been. For example, perceptions of sexual activity and risk may not have mirrored the ground realities of this study context, or individuals experiencing homelessness may have felt some inhibitions in fully reporting their activities via their phones. In either instance or with other potential explanations, people experiencing homelessness could guide the ideal length of a study period or ensure that data collection via phones is secure and anonymous. Ultimately collaborative engagement from community members is critical in evaluating how best to structure and implement this kind of data collection effort. Community stakeholders and partners can also work with investigators to further refine concepts of place. For example, study participants may have multiple places that either foster or inhibit risk. Fully understanding the places they experience daily, weekly or monthly, may be essential to truly gauge their risk.

In their study of PrEP access, Kim et al. also innovate around the concept of place. They note that definitions of residential neighborhoods have traditionally been static and constrained by administrative boundaries such as zip codes and census tracts [10]. They use global positioning technology (GPS) to define the places of activity of HIV-negative young men who have sex with men (MSM) living in New York City and within these places the presence of providers of Pre-Exposure Prophylaxis (PrEP). These researchers then measured the degree to which study participants had current or previous PrEP use. They found, after GPS monitoring of participants for 2 weeks, that PrEP accessibility within GPS-based defined activity space was positively associated with current or previous PrEP use [PR for activity space = 1.02, 95% CI (1.00, 1.03)] [10]. They also found that when using traditional administrative definitions of neighborhoods, the presence of PrEP providers in those spaces was not associated with PrEP use [PR for the residential area using census track = 1.09, 95% CI (0.88, 1.37); PR for the residential area using zip code = 0.99, 95% CI (0.93, 1.13)] [10]. Their innovative work suggests that meaningful assessments of place and access must reflect how individuals really live, move, and interact with their environment.

The research conducted by Kim et al. [10] highlighted additional important domains in designing, implementing, and evaluating research grounded in community space and activity. Indeed, research that tracks the movements of individuals who may experience stigma, as well as their possible interactions with health care providers, must be contemplated and constructed in close consultation with community partners. Potential risk must be balanced against the undeniable value of refining an understanding of neighborhoods and how individuals manage their environments. Community partners can also offer valuable insights into other spatial barriers or facilitators that individuals may encounter as they seek a PrEP provider. Transportation, clinic hours, or proximity to other regularly used services may also determine how and where an individual moves and accesses PrEP. Finally, interpreting and communicating the results of studies that map individual behavior and movement, such as those of Madden et al. and Kim et al. requires close collaboration between investigators and community partners. Like models, mapping research offers welcome precision and precious insights. Yet, just as with models, maps must be considered with an evident appreciation of what data is included and not across place and time.

## Measures

As in other work described in this volume, Blankenship et al. pushed the boundaries of our understanding of the social and structural determinants of HIV and how they operate at both a conceptual and methodological level [11]. Their work is framed in a social determination model as opposed to a social determinants model. They posit that the framing of social determinants as forces that are “upstream” impacting health outcomes that are “downstream” obscures the reality of HIV risk, which occurs within dynamic and inter-related structural forces and is often tied to historic and long-standing systems oppression. Their approach to the study of social determination is also innovative, drawing on longitudinal qualitative interviews synthesized into case studies that examine the experiences of women living in low-income, predominantly racial/ethnic minority neighborhoods. The interviews, which were conducted every 6 months for 2 years, demonstrated in a way that cross-sectional qualitative work couldn’t, how HIV prevention and mitigation of sexual risk occurs within a dynamic and relentlessly challenging context of housing vulnerabilities, mass incarceration, and state-led surveillance, including criminal justice and social service systems. Significantly, these case studies and the insights they provide come from the community, with investigators serving to document and witness a foundational reality.

The focus on community voices and experiences and how these experiences evolve in this work is an important innovation in the measurement of social and structural factors and an exemplar of the need to let the community tell the story. At the same time, there is space for further collaboration. Theoretical and conceptual models of social determinants of health and social determination can be formed and generated around lived experience. We can look to individuals who have navigated the barriers presented by social determinants and social determinism and ask them which models resonate and whether they have thoughts on what the ideal conceptual or theoretical understanding might entail. Further, while as Blankenship et al. [11] detailed, housing, incarceration, and dealing with social surveillance systems are central themes that underscore vulnerability to HIV, there may be other forces that need to be investigated and considered. Community partners and members can offer their perspectives on which forces are pre-eminent and suggest other forces of equal or more significant influence such as violence, gender norms, poverty, or educational access. In any case, at both a theoretical and operational level, full integration of community in conceptualizing and framing social determinism research is critical if we are to achieve an accurate and evolving understanding of how social determinants weave their influence in individual lives over time and how best to influence policies and programs that address these barriers.

In a final example of how the work contained in this volume aimed to both address gaps in the field around the measurement of social determinants as well as respond to the needs of the community, Kerrigan et al. describe the development of a measure that acknowledges the reality of intersecting social determinants including in this instance, occupational stigma among female sex workers (FSW) [12]. The Experiences of Sex Work Stigma (ESWS) scale reflects a mixed-methods, community grounded approach that was implemented across geographic and epidemic settings. The investigative team worked with FSW living with HIV in Tanzania and the Dominican Republic to identify scale domains through in-depth interviews and cognitive debriefing interviews. Participants defined how they experience stigma and which domains, such as shame, silence, and treatment received, resonated with this experience. Through this iterative, community-engaged work, a new domain of resisted stigma or dignity about sex work as an occupation emerged. A survey test of these domains and an item response theory (IRT) analysis led to the creation of context-specific domain scores, which acknowledged differences in how the scale functioned in each setting. A measure that reflects the thoughts and language of marginalized groups managing the challenges of structural determinants on a daily basis and that works across settings is an important innovation. Further, this tool, a reliable (ESWS > 0.80, with alphas ranging from 0.81 to 0.93) and valid (all domains were associated with at least one of the expected outcomes) scale that assesses multiple domains of sex work stigma, will enable both investigators and community alike, to better understand how experiences and resistance of sex work stigma are intertwined with internalized HIV stigma, depression, anxiety, sexual partner violence, and social cohesion.

The work of Kerrigan et al. [12] raised the central importance of context in the study of social determinants. As elegantly demonstrated in this work, how stigma is internalized and experienced and even perceived across settings can range from slightly nuanced to vastly distinct. It is possible, for example, that scores on a stigma measure designed and validated in a single setting may mean very different things when applied across settings. To be fully valid, the measurement of any social or structural determinant then must attend to context. This is most effectively done when research is based on an understanding of the context that reflects long-standing collaboration, partnership, and trust between investigators and the community. As with other work discussed in this volume, charting a course for research in the modeling, mapping, and measuring of social determinants is also best done in collaboration with community partners. The lived experience and pressing needs of individuals struggling with the obstacles presented by social determinants should guide research and programmatic imperatives. Further, the impact and success of research-driven policy and program development will best be evaluated with measures and assessments derived from the authentic insights of the very individuals these policies and programs aim to serve.

## Discussion

As this supplement comes to press, we are amid a global COVID-19 pandemic that has underscored what we already know well from the HIV global pandemic [13, 14]. That is that social determinants are again and again and again responsible for devastating inequities in morbidity and mortality among minoritized populations and the overall human toll of these pandemics. Throughout its history, the response to the HIV epidemic has demonstrated the importance of engaging the community in mitigating this toll, from advocacy for dedicated HIV funding to community-based participatory research to the national Ending the Epidemic (EHE) initiative, which takes a an important step towards mandating meaningful collaboration between academic investigative teams and community partners conducting implementation science work [15].

The approaches and conclusions discussed in this volume offer examples of a next step in the trajectory of this partnership where research and policy agendas are constructed collaboratively with academic, community, and federal partners. Importantly, these efforts must go beyond documenting inequities and developing better methodological approaches and interventions that reflect praxis and the imperative to disrupt the systems of oppression that cause and perpetuate these inequities [16]. The urgent need for this next step could not be more evident. We suggest that in the same way investment has been made in HIV vaccines via the HIV Vaccines Trial Network (HVTN) and clinical trials for prevention approaches via the HIV Prevention Trials Network (HPTN), so should similar investments be made in the socio-structural determinants of HIV. An HIV social determinants trials network to advance interventions that mitigate the impact of the social determinants through research that is entirely community-driven would generate benefit not only for those most vulnerable to HIV but for those who are most vulnerable across the landscape of infectious and chronic diseases. An important component of such a network would be the development of sophisticated and nuanced models, maps, and measures that are founded in community experience including intersectional and interconnected identities and social positions, evaluated with criteria that reflect that authentic experience and that support multi-sectoral efforts towards policy and program change that dismantle socio-structural systems of oppression.

As mentioned above, a concrete and innovative example of how academic, community, and federal partners are moving the HIV research agenda forward is the EHE initiative. This body of work reflects a significant investment aimed at ensuring that the full benefit and promise of advances in biomedical prevention such as PrEP or long-acting treatment is realized. The field of implementation science is expanding rapidly and speaks directly to the need to fully respond to the role of social determinants as important challenges and barriers to accessing and engaging in innovations in prevention and treatment. Work such as that discussed here that is community-driven and that maps, models, and measures the socio-structural determinants of health must be woven into implementation science approaches that fully confront longstanding structural inequities built by the policies and structures of agencies and institutions. Much as implementation science is needed to ensure the success of biomedical advances in HIV, so is a comprehensive appreciation of socio-structural determinants and community contexts required to ensure the success of implementation science efforts that disrupt systems of oppression.

Finally, again in this complex moment both nationally and internationally, we see the limitations of a traditional model of combatting HIV, COVID-19, or any other emergent disease. Academic and federal partners are developing an increasingly nuanced understanding of how structures and systems such as those that define how epidemics are managed or health care is delivered or how academic research is structured and conducted are inadequate and outdated. As we collectively delve into historically oppressive systems with colonial antecedents, we must be ready to challenge these systems and replace them with collaborative models. Community partners should drive the agenda and fully engage in all aspects of the study design, including models, maps and measures, study implementation, data interpretation, and the dissemination of findings. A meaningful reduction of the impact of the HIV epidemic or other deadly diseases is only possible with this kind of collaborative practice.
